# Corneal Confocal Microscopy in Postural Orthostatic Tachycardia Syndrome (POTS) as a Diagnostic Tool for Small Fiber Neuropathy

**DOI:** 10.7759/cureus.82781

**Published:** 2025-04-22

**Authors:** Christopher Cantrell, Ryan Rilinger, Samantha J Stallkamp Tidd, Robert Wilson

**Affiliations:** 1 Cleveland Clinic Lerner College of Medicine, Cleveland Clinic, Cleveland, USA; 2 Neurology, University of Rochester, Strong Memorial Hospital, Rochester, USA; 3 Neurology, Cleveland Clinic, Cleveland, USA

**Keywords:** ccm, ccmetrics, corneal confocal microscopy, nerve branch density, nerve fiber density, nerve fiber length, postural orthostatic tachycardia syndrome, pots, sfn, small fiber neuropathy

## Abstract

Objective: Postural orthostatic tachycardia syndrome (POTS) is a debilitating condition characterized by autonomic dysregulation. Patients with this disorder may experience orthostatic intolerance, palpitations, fatigue, and a wide variety of other symptoms. The neuropathic symptoms of POTS may be caused by small fiber neuropathy (SFN), which is currently diagnosed using skin nerve biopsy. Corneal confocal microscopy (CCM) is an imaging modality that allows visualization of the corneal nerve layer. Our study aimed to determine whether CCM could detect differences in small nerve fiber parameters between POTS patients with and without signs or symptoms of SFN.

Materials and methods: CCM was performed on nine patients, along with a neurological examination. Participants were also asked about neuropathic symptoms by a researcher. Based on examination findings and/or reported symptoms, patients were categorized into SFN+ and SFN- groups for comparison. A chart review was conducted to gather demographic data, medications, autonomic testing results, and medical history, including common POTS comorbidities.

Results: Comparison of nerve fiber parameters using CCM did not reveal a statistically significant difference between the groups. However, valuable insights were gained regarding the logistics of conducting this type of study in POTS patients, including adapting to challenges and improving coordination between the neurology and ophthalmology departments.

Conclusions: CCM may one day replace skin nerve biopsy as a diagnostic tool for SFN in POTS patients. Although this preliminary analysis did not demonstrate significant findings, likely due to the small sample size, we believe CCM may still have a role in POTS research and could eventually become a diagnostic tool used in autonomic clinics.

## Introduction

Postural orthostatic tachycardia syndrome (POTS) disrupts the autonomic nervous system, inducing excessive heart rate increase upon standing without concurrent orthostatic hypotension [1,2]. The collection of symptoms experienced by POTS patients is heterogeneous, resulting in a wide range of presentations for this disorder [3-5]. The hallmark symptoms are cerebral hypoperfusion-induced presyncope or syncope as well as palpitations, with symptoms quickly improving upon recumbence [1,4]. Among the other symptoms suffered by POTS patients are chest pain, headache/migraine, brain fog, light sensitivity, nausea, constipation/diarrhea, bladder dysfunction such as polyuria, fatigue, exercise intolerance, and neck pain [2,6,7]. First-line treatments for POTS include lifestyle management, such as exercise, compression stockings, and increasing salt intake, and off-label medications like beta-blockers and fludrocortisone. However, there are still no FDA-approved treatments for POTS [8,9]. New treatments are generally targeted toward specific symptoms at this time, including medications like low-dose naltrexone [10].

It is estimated that POTS affects around 1-3 million people in the U.S., though the prevalence has not yet been definitively researched [1]. Females much more frequently have POTS than males (the female-to-male ratio is above 4:1), and those with this condition are typically younger than age 50 [1,3,4]. About 90% of diagnosed POTS patients are White individuals, and symptom onset is usually reported during the teenage years [2]. Inciting factors for POTS are variable and sometimes may not be identified at all [6,7]. Antecedent viral illness (usually gastrointestinal or upper respiratory tract, including COVID-19) is suspected in perhaps half of POTS patients, with other acute stressors that may precede POTS being major surgery and pregnancy [1,6,11,12].

Delay to diagnosis is frequently substantial for POTS patients, ranging from six months to three years in many cases [13,14]. Three-quarters of POTS patients report misdiagnosis before their POTS was discovered [15]. The most frequent misdiagnoses include anxiety, vasovagal syncope, chronic fatigue syndrome, and panic attacks [16,17]. These experiences can instill mistrust of physicians and the health care system, representing another barrier to receiving the care POTS patients need [14]. Combined with their symptoms, especially orthostatic intolerance and fatigue, this can limit function in day-to-day life and the ability to participate in aspects of their care [16].

Over half of POTS patients have impaired function of the peripheral sympathetic nervous system [7]. Neuropathic manifestations of POTS include decreased sweating and venous blood pooling in the legs due to insufficient peripheral vasoconstriction, which prevents the patient from maintaining blood pressure in the upper body upon standing [7,18]. In POTS patients with neuropathic symptoms, skin nerve biopsy can show decreased intraepidermal nerve fiber density (NFD) indicative of small fiber neuropathy (SFN) [19,20]. Small nerve fibers are responsible for somatic and autonomic processes, including pain, temperature sensation, sweating, vasoconstriction, and regulating functions such as those of the gastrointestinal system and bladder [21,22]. Symptoms of SFN include burning pain, hyperesthesia, allodynia, and shooting pain [23].

Corneal confocal microscopy (CCM) is an imaging modality that allows for the visualization of small nerve fibers inside the eye's lens [24]. In the past, CCM has been used primarily by ophthalmologists to diagnose *Acanthamoeba* infections and problems with the epithelium or stroma of the eye [25-27]. The cornea has the densest collection of small nerve fibers in the body [24]. As C-fibers, these small nerve fibers of the cornea are similar in structure to those that innervate the skin [24]. It has been shown that CCM can detect signs of SFN in diabetic patients, in addition to other conditions like fibromyalgia, post-COVID-19 neuropathy, and multiple sclerosis [24,28,29]. No studies have been published evaluating CCM's potential to be a less invasive alternative for skin nerve biopsy as a diagnostic tool for SFN in POTS patients.

This study aimed to investigate whether CCM detects a difference in nerve fiber parameters (nerve fiber length (NFL), NFD, and nerve branch density (NBD)) between POTS patients with and without symptoms and exam signs of SFN.

## Materials and methods

Inclusion and exclusion criteria

Patients could be included in our study only if they were seen by a neurologist at our institution, had a diagnosis of POTS, were between the ages of 18 and 50, had a positive tilt table test without orthostatic hypotension, had POTS symptoms for at least six months, and had a workup ruling out other causes of sinus tachycardia. Potential study participants were ruled out from participation eligibility if they had any of the following: other cause of neuropathy, history of chemotherapy treatment, history of eye surgery/trauma, concerning eye-related symptoms, use of orthokeratology lenses, current or previous corneal disease, or feeling unable to sit still during the imaging process or a neurological exam.

This study was conducted from September 22, 2023, to April 30, 2024. The Cleveland Clinic Institutional Review Board issued approval 20-240. All nine patients who participated in the study met the inclusion criteria and had no elements that excluded them. The tilt table test is the current standard for POTS diagnosis, which involves measuring a patient’s heart rate and blood pressure when they are supine compared to when they are tilted upward at a 70° angle from the horizontal plane. Supine heart rate and blood pressure, averaged over five minutes, are compared to readings recorded every minute with the table tilted. A positive tilt table test is defined by a sustained increase in heart rate of 30 beats per minute or more in the absence of orthostatic hypotension (decrease in systolic blood pressure of 20 mmHg or decrease in diastolic blood pressure of 10 mmHg compared to supine average) [1,30].

Recruitment process

First, POTS patients seen in our neuromuscular clinic were screened in the electronic medical record for age and a positive tilt table test. A total of 103 recruitment messages were sent via the electronic medical record to patients who met the inclusion criteria. Of those who received recruitment messages, 24 patients showed interest in the study, and 20 completed a phone call to participate in the recruitment. Fourteen study subjects were scheduled for a research visit, and five of them canceled. By the end of the study, nine research visits were completed.

Group assignment

To sort patients into groups for analysis, we designed a scoring system that incorporates both patient-reported symptoms and neurological examination findings (SFN+/- scoring system). During their research visit, patients underwent a neurological examination. They were asked about four neuropathic symptoms that they may or may not be experiencing: paresthesia, weakness/imbalance, neuropathic pain, and skin color changes. Patients who reported at least two symptoms of SFN and had at least two findings of neuropathy on the sensory component of the neurological exam would be assigned to the SFN+ group. If a patient did not meet the requirements for both neuropathy symptoms and exam findings, they were placed in the SFN- group. An additional requirement for the exam findings aspect of the SFN+/- scoring system was that one or more symptoms had to be specific to SFN, such as changes in pain and/or temperature sensation. Thus, for example, if a subject reported neuropathic pain and paresthesia and their exam was notable for loss of pain and temperature sensation, they would be sorted into the SFN+ group. If a patient has neuropathic pain and skin color changes but no neuropathic findings on examination, they would be assigned to the SFN- category.

Research visit details

Research visits included a comprehensive neurological examination. The four neuropathic symptom questions used for our SFN+/- scoring system were asked during the neurological exam. An ophthalmologist conducted a screening slit lamp exam to rule out any corneal abrasions or structural abnormalities that might make the CCM imaging process unsafe. CCM imaging was performed by a professional photographer using the HRT3 RCM in vivo corneal confocal microscope (Heidelberg Engineering Inc., Franklin, MA, USA). This microscope uses high magnification to detect a one-cell-thick layer of small nerve fibers in the cornea. The lens nearly touches the eye, and in some cases, it may make light contact with the cornea. A layer of gel between the lens and the subject’s eye is meant to keep the eye from drying out during imaging. Just before imaging, numbing drops of lidocaine were applied to the patient’s eyes to reduce irritation to the cornea. Each subject’s left and right corneas were imaged to acquire three quality images for analysis from each eye for a total of six pictures per patient [31].

Image analysis

**Figure 1:**
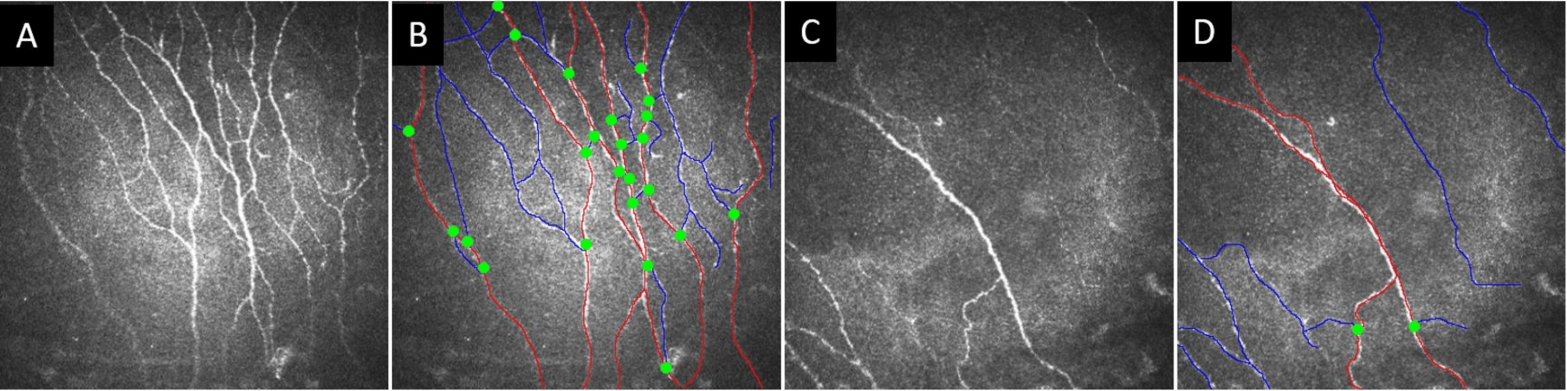
Raw and traced CCM images (high and low density) Examples of raw CCM images captured by the corneal confocal microscope and traced images by the CCMetrics program. On the left (A, C) is the raw image collected on the professional photographer's corneal confocal microscope, and on the right side (B, D) is the traced image after analysis in CCMetrics. To represent the variety of NFD in CCM images, the top images show higher nerve density, and the bottom images show an example of lower nerve density. The red lines designate nerve fibers, the blue lines identify nerve branches, and the green dots mark branch points from the nerve fiber. CCM: corneal confocal microscopy, NFD: nerve fiber density

The corneal confocal images were traced using CCMetrics software (University of Manchester, Manchester, UK). This program is designed to help users identify nerve fibers and nerve branches through manual tracing. Based on the tracing, it measures three nerve fiber parameters: NFD in fibers per mm², NBD in branches per mm², and NFL in mm per mm². After quantification, to evaluate for a difference in nerve fiber parameters between the SFN+ and SFN- groups, we used a Student’s t-test for comparison of the means of each parameter separately. Below are two example sets of CCM images with and without manual tracing overlay (Figure 1).

Chart review

The retrospective chart review component of this study involved collecting data on age, sex, race, past medical history, history of diabetes, medications, and family history. For tilt table findings, one of our inclusion criteria, numerical differences in heart rate and blood pressure, were collected. For the remaining three autonomic tests, the results were recorded as either normal or abnormal. Quantitative sudomotor axon reflex testing (QSART) evaluates the sweat response after skin stimulation with acetylcholine. If sweat production is lower than a specified threshold adjusted for sex and age, then the QSART for that patient is abnormal, suggesting dysfunction of postganglionic sympathetic nerve fibers [32]. The deep breathing test is performed by measuring the heart rate while breathing deeply in a supine position. This evaluates vagal nerve function and determines if respiratory sinus arrhythmia exists [20,33]. The Valsalva autonomic test induces increased intrathoracic pressure by having the patient close their airway while attempting to exhale. The baroreceptor reflex can be activated by a lower preload for the heart, allowing for evaluation of the heart's response and comparison to reference values [33]. The variables included in our chart review are categorized in Table 1. Notably, not all patients had every value available for collection.

**Table 1:** Variables collected in chart review Data for each of the variables listed were collected from each patient’s electronic medical record in EPIC. All gathered data was stored in the REDCap secure research database. Data stored but not presented in this thesis or used in analyses: BMI, date of initial visit to our autonomic center, date of symptom onset (if known), antecedent viral illness or TBI (if documented), past medical history, mast cell diagnosis, medications, allergies, family history, POTS symptoms on initial visit, neurological exam findings on initial visit, date of tilt table test, time of tilt syncope, date of QSART, date of skin punch biopsy, PROMIS global physical health T-score, and PROMIS global mental health T-score SBP: systolic blood pressure, DBP: diastolic blood pressure, QSART: quantitative sudomotor axon reflex testing, REDCap: Research Electronic Data Capture, BMI: body mass index

Variable	Definition
Age	Age in years of the patient on the day of their research visit
Sex	Biological sex as reported in the electronic medical record
Race	Race of the patient as recorded in the electronic medical record
Diabetes diagnosis	Whether the patient has a diagnosis of diabetes, which would be concerning as a confounding factor for any neuropathic symptoms
Tilt supine heart rate	Baseline heart rate for the tilt table test, averaged over 5 mins
Tilt maximum heart rate	Maximum heart rate on head-up tilt during tilt table test
Tilt table heart rate change	Equals the tilt maximum heart rate minus tilt supine heart rate
Supine SBP	Baseline SBP for the tilt table test, averaged over 5 mins
Supine DBP	Baseline DBP for the tilt table test, averaged over 5 mins
Tilt table max SBP change	Max decrease in SBP during head-up tilt compared to supine SBP
Tilt table max DBP change	Max decrease in DBP during head-up tilt compared to supine DBP
Syncope on tilt	If the patient had syncope or not during the tilt table test
QSART result	Whether the patient had a normal or abnormal sweat response during the QSART test
Valsalva test result	Whether the patient had a normal or abnormal heart rate/blood pressure response during the Valsalva test
Deep breathing test result	Whether the patient had a normal or abnormal heart rate response during the deep breathing test
Skin nerve biopsy result	If there was a normal or abnormal result on the skin nerve biopsy (if the patient had a biopsy in the past)

Skin nerve biopsy data were collected from the three patients who had previously undergone the procedure during their workup for neuropathic symptoms. In our autonomic center, a pair of 3-mm biopsies is taken from the patient’s leg, one from the distal thigh and the other from the distal lower leg. The laboratory then assesses intraepidermal NFD, and an abnormal classification is assigned if the NFD is less than the fifth percentile [20].

## Results

Patient characteristics and group classifications

The average age of the nine patients in our study was 33.67 years, with a standard deviation of 9.17. Our study sample was 89% female and 78% White individuals. None of our patients had a diagnosis of diabetes, which could have been a confounding comorbidity. There were four patients in the SFN+ group and five in the SFN- group. Tilt table testing showed a mean maximum heart rate increase of 42.22 beats per minute over the first 10 minutes of head-up tilt. Valsalva and deep breathing tests were normal for each patient on record, while three out of four patients had abnormal QSART results. Only three patients had skin biopsy testing done as part of their SFN workup, and one of those samples returned with abnormal intraepidermal NFD (Table 2).

**Table 2:** Patient characteristics and group classification SD: standard deviation, SFN: small fiber neuropathy, SBP: systolic blood pressure, DBP: diastolic blood pressure, QSART: quantitative sudomotor axon reflex testing

Patient characteristics (n=9)	
Age (years, mean ± SD)	33.67 ± 9.17
Sex (female, n(%))	8 (89%)
Race (White, n (%))	7 (78%)
Diabetes diagnosis (yes, n(%))	0 (0%)
Group classification	
SFN+ group (n(%))	4 (44%)
SFN- group (n(%))	5 (56%)
Tilt table testing	
Tilt supine heart rate (bpm, mean ± SD)	82.44 ± 13.45
Tilt maximum heart rate (bpm, mean ± SD)	124.67 ± 12.67
Tilt table heart rate change (bpm, mean ± SD)	42.22 ± 6.40
Supine systolic blood pressure (mmHg, mean ± SD)	116.11 ± 19.05
Supine diastolic blood pressure (mmHg, mean ± SD)	72.11 ± 11.67
Tilt table max SBP change (mmHg, mean ± SD)	-2.89 ± 7.69
Tilt table max DBP change (mmHg, mean ± SD)	-1.67 ± 6.38
Syncope on tilt (yes, n(%))	1 (11%)
Other autonomic testing	
QSART result (abnormal, n/(# of patients with test result) (%))	3/4 (75%)
Valsalva test result (abnormal, n/(# of patients with test result) (%))	0/6 (0%)
Deep breathing test result (abnormal, n/(# of patients with test result) (%))	0/5 (0%)
Skin nerve biopsy result (abnormal, n/(# of patients with test result) (%))	1/3 (33%)

Nerve fiber parameters by SFN+/- group

**Figure 2:**
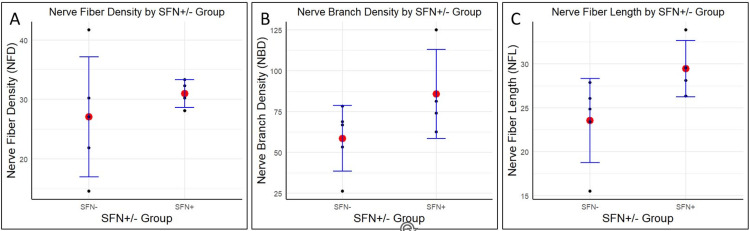
Nerve fiber parameters by SFN+/- group For each of the three nerve fiber parameters (NFD in fibers/mm2 (A), NBD in branches/mm2 (B), and NFL in mm/mm2 (C)), the values generated from manual tracing in CCMetrics were plotted for the SFN+ and SFN- groups. Red dots designate the mean for a measured variable in that group, and smaller black dots represent individual patient values to show the distribution. Five patients are in the SFN- group and four in the SFN+ group. No statistical significance was based on Student’s T-tests for each variable compared between the SFN+ and SFN- groups (means, SDs, and p-values provided in text above). SFN: small fiber neuropathy, NFD: nerve fiber density, NBD: nerve branch density, NFL: nerve fiber length, SDs: standard deviations

None of the three analyzed nerve biopsy parameters (NFD, NBD, NFL) demonstrated a significant difference between the SFN+ and SFN- groups. Mean NFD for the SFN- group (mean=27.08, SD=10.07) did not show a significant difference (p=0.443) compared to the SFN+ group (mean=30.99, SD=2.31) (Figure 2A). This was also the case (p=0.154) for NBD compared between those who were SFN- (mean=58.54, SD=20.24) and SFN+ (mean=85.68, SD=27.33) (Figure 2B). NFL averages were not significantly different (p=0.063) for the SFN- (mean=23.53, SD=4.78) and SFN+ (mean=29.45, SD=3.20) groups (Figure 2C).

## Discussion

This study aimed to evaluate whether CCM can detect differences in nerve fiber parameters (NFD, NBD, NFL) between patients with and without signs and symptoms of SFN. As an initial investigation of CCM in the context of POTS, we approached this research with an exploratory mindset, understanding that a preliminary analysis might not provide a complete picture of this technology’s potential as a diagnostic tool.

The demographics of our patient sample were similar to those of other POTS studies. Our cohort's average age was 33.67 years. The study population consisted of 89% females and 78% White individuals. Recent publications from our institution have reported an average age of 27-36 years, with 81.25-92% female and 87.9-93.75% White individuals [8,12,19,34]. These average ages fall within the expected range for most POTS patients, and the vast majority being female is consistent with what is expected [1,35]. In addition, it was expected that autonomic testing other than tilt table (QSART, Valsalva, deep breathing) would not be positive for all patients, as this has been reported in a comprehensive chart review of POTS patients seen at our institution [19]. POTS patients are known to be a heterogeneous population in terms of symptoms and autonomic testing, which is reflected in our study sample.

Comparing nerve fiber parameters between groups did not reveal any statistical significance. Interestingly, the mean NFD, NBD, and NFL were higher in the SFN+ group than in the SFN- group. However, we believe this is most likely due to the low outlier in the SFN- group, which significantly lowers the mean for that subset. With only nine patients successfully recruited and imaged, this analysis had a too small sample size to draw definitive conclusions about CCM’s ability to detect differences in NFD, NBD, and NFL in POTS patients.

CCM, if validated in larger future studies, could have multiple benefits for POTS patients with SFN. As a noninvasive imaging modality, CCM avoids the multiple scars left on the patients’ legs from a punch biopsy. It is possible that CCM could be a more sensitive test than skin nerve biopsy, given the higher density of small nerve fibers in the cornea compared to the skin [24]. If validated, CCM could benefit POTS patients in both improved likelihood of an accurate neuropathy diagnosis and reduced invasive testing burden. In addition, if it is found to be reliable in detecting small nerve fiber differences in POTS, CCM would enhance our ability to study SFN over time in our patients. Few patients would be interested in serial skin biopsies, but we expect that more would be willing to participate in corneal imaging every one to two years to track their SFN status over time. In this study, only one adverse event was recorded: a patient experienced lingering eye irritation after CCM imaging, which resolved the following day.

The ideal study to assess the value of CCM for SFN diagnosis in POTS would directly compare CCM to the results of skin nerve biopsy. If each subject had both a skin nerve biopsy and CCM completed, we could perform a direct comparison between patients. However, not many patients have a skin nerve biopsy done as part of their POTS workup. Adding a skin nerve biopsy to the protocol would create another barrier to recruitment. This kind of prospective study would likely have to run over several years or involve multiple centers. It would also be best to include a healthy control group without POTS, who would undergo skin punch biopsy and CCM. A healthy control group would provide an optimal comparison, based on baseline values of corneal nerve fiber parameters and the results of a skin nerve biopsy. Therefore, our study can serve as a blueprint for future studies to build upon. The methods employed in our study can serve as a helpful guide for the POTS research community as they seek to investigate the potential of CCM further.

To summarize our limitations, sample size was the primary constraint. A low rate of progression from recruitment to complete research visits impaired our ability to reach our sample size goal. The second major limitation was the subjectivity involved in patient group assignment, which, since sensory symptoms were patient-reported for both the neurological exam and symptom questions asked during the research visit, could not be avoided with this study design. Even a clinical diagnosis of sensory-predominant neuropathy can be subjective, so relying on a past diagnosis by a neurologist would not have solved this problem [36]. It is also important to note that not every patient had every variable collected in the chart review. Another limitation of our study was that we did not control for medications or comorbidities. In anticipation of potential recruitment difficulties, we recruited patients who met the inclusion and exclusion criteria. In addition, as a preliminary look into CCM as a tool for SFN evaluation in POTS, we chose to examine the POTS population seen at our tertiary care center broadly, regardless of their medications or comorbidities. Future studies could delve deeper into factors such as treatment regimens and comorbidities that may help refine our interpretation of the varying corneal nerve fiber parameters seen in POTS patients.

Next steps for this research work are crucial to capitalize on the framework established by this preliminary analysis. An increased sample size would allow for more comprehensive analyses. Ideally, future prospective studies would directly compare CCM results to skin nerve biopsy within the same patient. Although the invasiveness of the biopsy would be a barrier to increasing the study sample size, recruiting patients who already had skin nerve biopsies performed during their clinical care would improve feasibility.

## Conclusions

POTS is a debilitating condition of dysautonomia that can present with neuropathic symptoms in some patients. The relationship between POTS and SFN is well-established, but the diagnosis of SFN is currently either clinical or through skin punch biopsy. Based on prior research with CCM in diabetes and other conditions, we believe that CCM could benefit POTS patients as a diagnostic tool for SFN. The results described in this study represent a preliminary analysis. CCM did not identify statistically significant differences in nerve fiber parameters between patients with and without signs and symptoms of SFN. However, this paper still represents a novel research framework for investigating CCM in POTS patients, with the potential to guide future, larger-scale, prospective studies. Neuropathic symptoms depend on patient-reported outcomes and are inherently subjective, but they also provide insight into how patients experience their condition. With a larger sample size and consistent improvement in evaluating patient-reported outcomes, the unclear association between POTS and SFN can be better understood. What will be most important is whether, in the clinic or during a research visit, we, as physician-scientists, lead with listening and provide these patients with the care they deserve.
